# Circulating extracellular vesicles protein expression for early prediction of platinum-resistance in high-grade serous ovarian cancer

**DOI:** 10.1038/s41388-025-03382-4

**Published:** 2025-04-10

**Authors:** Vincent Wagner, Molly Morton, Kalpana Deepa Priya Dorayappan, Anna Gonzalez, Lianbo Yu, Takahiko Sakaue, Thomas Conrads, G. Larry Maxwell, Casey Cosgrove, Floor Backes, Qi-En Wang, David E. Cohn, David M. O’Malley, Karuppaiyah Selvendiran

**Affiliations:** 1 Division of Gynecologic Oncology, Holden Comprehensive Cancer Center, The University of Iowa Health Care, Iowa City, IA USA; 2 Division of Gynecologic Oncology, The James Comprehensive Cancer Center, The Ohio State University, Columbus, OH USA; 3 Department of Biomedical Informatics, The Ohio State University, Columbus, OH USA; 4 Division of Gastroenterology, Department of Medicine, Kurume University School of Medicine, Kurume, Japan; 5 Inova Women’s Service Line and the Inova Schar Cancer Institute, Falls Church, VA USA; 6 Department of Radiation Oncology, The James Comprehensive Cancer Center, The Ohio State University, Columbus, OH USA

**Keywords:** Cancer, Biomarkers

## Abstract

Platinum resistance in high-grade serous ovarian carcinoma (HGSOC) portends a poor prognosis. Although initial platinum-based chemotherapy response rates are high, 15-20% of patients demonstrate primary resistance to platinum therapy and almost all patients will develop platinum resistance in the recurrent setting. No predictive or diagnostic biomarkers have been utilized specific to platinum resistance. This study aimed to identify candidate biomarkers for platinum resistance in HGSOC using an extracellular vesicle (EV) based approach. We found differentially expressed and distinct EV proteins, namely TMEM205 and CFH, in patients with platinum-resistant (PR) HGSOC compared to those of platinum-sensitive (PS) patients, utilizing liquid chromatography-tandem mass spectrometry (LC-MS/MS). Expression of these EV proteins were validated in patient-derived PR cell lines as well as in clinically relevant mouse models of HGSOC post-platinum therapy. We corroborated these findings using serum samples from patients with PS and PR-HGSOC. Both EV CFH and EV TMEM205 exhibited excellent diagnostic capability for PR as noted by receiver operating characteristic curves with area under the curve values of 0.95 and 0.84, respectively. The high diagnostic performance of TMEM205 and CFH within EVs compared to the relatively poor performance of conventional serum proteins such as Ca125 suggests their robust potential as non-invasive biomarkers for detecting platinum resistance in HGSOC. Furthermore, the ROC curve for the combined biomarker demonstrated excellent diagnostic performance, with an AUC of 0.973, a true positive rate (TPR) of 0.938, and a false positive rate (FPR) of 0.062. Incorporating this multi-protein biomarker panel alongside established biomarkers further enhances diagnostic accuracy. Serum EV CFH and TMEM205 are promising biomarkers for early detection of platinum resistance in HGSOC and may highlight underlying chemoresistance mechanisms, offering potential future therapeutic targets.

## Introduction

Ovarian cancer continues to be the most lethal gynecologic cancer in the United States [1]. High-grade serous ovarian carcinoma (HGSOC), the most common histologic subtype, is associated with a poor prognosis as most patients are diagnosed at an advanced stage given the lack of effective screening methods [2–5]. While initial response rates to platinum-based chemotherapy are favorable in HGSOC, the majority of patients eventually develop resistance to platinum-based chemotherapy [2–5]. Patients with platinum-resistant (PR) HGSOC have a median survival of only 7–12 months [6–9]. The clinical response rate to non-platinum-based chemotherapy for patients with recurrent HGSOC is a mere 15–40% [10, 11]. To overcome the poor prognosis that platinum resistance portends and minimize the use of ineffective therapy, it is imperative to evaluate potential biomarkers associated with platinum resistance in HGSOC. Clinically, CA125 remains the standard biomarker for disease monitoring and evaluation of treatment response, however it is not sensitive nor specific enough to predict when a patient is or will become resistant to platinum-based chemotherapy [12–15]. Therefore, with this study, we sought to discover biomarkers that can more precisely predict and identify platinum resistance in the treatment of HGSOC.

Many proteomic biomarkers for ovarian cancer have been identified, however the vast majority have failed to be validated or adopted into clinical practice. This is likely due to the high complexity of the serum proteome. The serum contains a broad range of highly abundant proteins making the detection of less abundant cancer-specific biomarkers more difficult to reliably detect, especially in low-volume disease states [16, 17]. In contrast to serum proteins, extracellular vesicles (EVs) are an attractive source of cancer-specific biomarkers as they carry cell-specific cargo (proteins, microRNAs, and lipids) from their cells of origin, are highly stable, and can be obtained from any biological fluid using non-invasive methods [18–20]. EVs are nano-sized vesicles ranging from 30 to 120 nm that originate within the endosomal system and are secreted by a diverse array of cells. EVs sourced from tumors have gained prominence as potential biomarkers due to their ubiquitous presence in various bodily fluids and unique characteristics that distinguish them from non-cancer cell-derived EVs. EVs also have a less complex proteome compared to whole serum, allowing for easier detection of cancer-specific proteins [21–23]. Recent investigations have highlighted the potential of circulatory EV-associated microRNA and proteins as valuable biomarkers for a spectrum of cancer types [24, 25]. There is growing evidence of elevated EV secretion from platinum-resistant ovarian cancer cells [26, 27]. EV-derived microRNA, such as miR-21-3p, has been demonstrated to play a role in inducing platinum resistance in previously platinum-sensitive (PS) ovarian cancer cells [28]. These findings underscore the evolving landscape of EVs as a reservoir of diagnostic and mechanistic insights in the study of platinum-resistant ovarian cancer. Although EVs exhibit significant promise and versatility as biomarkers of malignancy, no specific biomarkers for PR-HGSOC have been identified. In our current scientific investigation, we endeavor to identify potential biomarkers associated with PR-HGSOC through a comprehensive analysis of EV proteins.

## Materials and methods

### Cell lines and animals

The study employed immortalized high-grade serous ovarian cancer (HGSOC) cells, including platinum-sensitive OVCAR-3 and PEOC1, along with platinum-resistant patient-derived ascites cells (R127, & R182) provided by Dr. G. Mor of Yale University. These cells originated from patients with recurrent, platinum chemotherapy-resistant ovarian cancer. To maintain experimental integrity, mycoplasma activity in all cell lines was assessed bi-monthly using the ATCC® Universal Mycoplasma Detection Kit. Thawed cells underwent a maximum of 5 passages, after which they were discarded, and a new vial was utilized. For in vivo experiments, HGSOC cells (3 × 10^6^ cells in 100 μl of PBS) were injected into the ovarian bursa of 6-week-old BALB/c nude mice from the OSU Transgenic Mice Core Lab (*N* = 10). Periodic in vivo MRI imaging tracked tumor growth. Upon sacrifice, tumor weight and volume were measured. The animal protocol adhered to ethical standards, approved by OSU IACUC (Protocol number 2012A00000008-R3), ensuring humane treatment in accordance with vertebrate ethical regulations.

### Procurement, processing and handling of human samples

Retrospectively collected platinum sensitive and resistant ovarian cancer patient samples were obtained from The Ohio State University James Cancer Hospital and Solove Research Institute (*N* = 22), (IRB study number— 2021C0031) and Inova Schar Cancer Institute (*N* = 32). Patients were assessed by physician review of clinical records at the time of serum collection and were considered to be platinum resistant if they experienced progression of disease during treatment with a platinum-based chemotherapy regimen or progression of disease within 6 months from last platinum exposure (<6 month platinum free interval). Patients were otherwise considered platinum sensitive. Deidentified patient information including age, diagnosis, histology, grade, germline genetics, phase of treatment, and disease distribution was obtained by retrospective review of clinical records. For the controls, samples were included if the patient had no documented adnexal pathology, cancer, or comorbidities. The patient serum samples were stored at −80 °C and were subjected to thawing at 30–50 min prior to experiments.

### EV isolation from culture medium

To isolate tumor EVs from cultured media, immortalized ovarian cancer cells were cultured for 48 h in medium with EVs-depleted FBS (System Bioscience, CA, US). The conditioned medium underwent sequential centrifugation steps (400 × *g* for 10 min, 10,000 × *g* for 30 min) and filtration through 0.22 μm membranes. EVs were then isolated via ultracentrifugation at 100,000 × *g* for 1 h at 4 °C, followed by a wash in 1× PBS and a second ultracentrifugation step. The final pellet was re-suspended in PBS, and vesicular protein concentration was determined using the BCA protein assay (Thermo Fisher Scientific). For patient samples, frozen serum and fresh ascites were used with approval from the Ohio State University IRB (Study number 2004C0124). EVs from 250 μl of cell-free serum were isolated following a similar protocol as for cell culture medium. The resulting EVs pellet was re-suspended in 100 µl of cold PBS or lysis buffer for downstream applications, including NTA, TEM, and Western blotting, as previously described [29].

### EV isolation from mice and patient serum samples and evaluation of EV proteome profile in platinum sensitive and resistant HGSOC serum samples by LC–MS/MS: Exosome isolation by commercial kit

The EVs were isolated from HGSOC or control patient serum samples (100 μl) using the Exo-quick kit solution according to the manufacturer’s (System biosciences) protocol. The resulting exosome pellet was re-suspended in either 100 µl of cold PBS or lysis buffer, depending on the downstream application. **Isolation of EV using SEC columns:** IZON qEV original size exclusion columns (Izon Science) were used in the isolation of EV. The columns were first removed from 4 °C and the 20% ethanol storage solution was allowed to run through the column followed by 20 ml particle-free PBS. Serum samples were diluted to 500 µl with sterile filtered particle-free PBS and the sample was overlaid on the qEV size exclusion column followed by elution with particle-free PBS. The flowthrough was collected in 500 µl fractions, and fractions 1–4 were pooled for further downstream analysis such as Image stream flowcytometry. For ELISA plating the pooled EV samples were further concentrated using an Amicon Ultra-0.5 ml (10 kDa) centrifugal filter device (Merck Millipore) for protein estimation and relative quantification of potential EV biomarker proteins across different patient samples.

Proteins extracted from EVs were digested with trypsin and the digests were analyzed using LC-MS/MS for protein identification and quantitation. Briefly, samples were first separated on an EASY Spray PepMap C18 column (3 μm, 100 Å, 0.75 × 150 mm Thermo Scientific) coupled to a Dionex UltiMate 3000 RSL Cnano HPLC system. The peptides eluted from the column were analyzed on a Thermo Fisher Scientific Orbitrap Fusion mass spectrometer operated in positive ion mode using data-dependent Top Speed method.

### EVs protein quantification and analysis: ELISA

EVs were lysed using RIPA buffer added with protease cocktail inhibitors and incubated at room temperature for 5 min. Protein quantification was performed using the direct detect infrared spectrophotometer or NanoDrop UV-Vis spectrophotometer. The plates were coated with equal concentration of EVs proteins and incubated on a 96-well assay plate for 48 h, and then probed with the optimized primary and secondary antibody concentration titrated for each protein target. On addition of the TMB substrate solution to the HRP-conjugated secondary antibody, blue color develops which corresponds to the target protein concentration. The reaction was then stopped by the addition of 0.2 N Sulphuric acid, which turns the reaction mixture to yellow color. Readings were obtained within 10 min at 405 nm.

### Statistical: sample size

With 10 patient samples per group, we achieved 80% power to detect a threefold difference in proteomic expression between groups, with coefficient of variation (CV) of 60%, at significance level $\alpha =\frac{0.05}{10\times 3}=0.0025$ adjusting for 10 candidate biomarkers and 2 contrasts (platinum-resistant HGSOC vs. platinum-sensitive HGSOC and controls). To minimize the bias caused by confounding covariates, stratified random sampling applied in selecting 10 platinum-resistant HGSOC patients from the available patients, and propensity score matching was performed in selecting platinum-sensitive HGSOC patients and controls matched to platinum-resistant HGSOC patients by the optimal method.

### Biomarker comparison between groups

For proteomics LC–MS/MS profiling, R package limma were used to compare the abundance of different groups by fitting through an empirical Bayes method with estimates weighted by the VOOM variance method of the log-counts. Differentially expressed (DE) proteins were selected using both fold-change and significance cutoff by controlling for the expected mean number of false positives.

## Results

### Isolation of EVs and identification of initial candidate proteins in PR-HGSOC patient serum samples

**Fig. 1:**
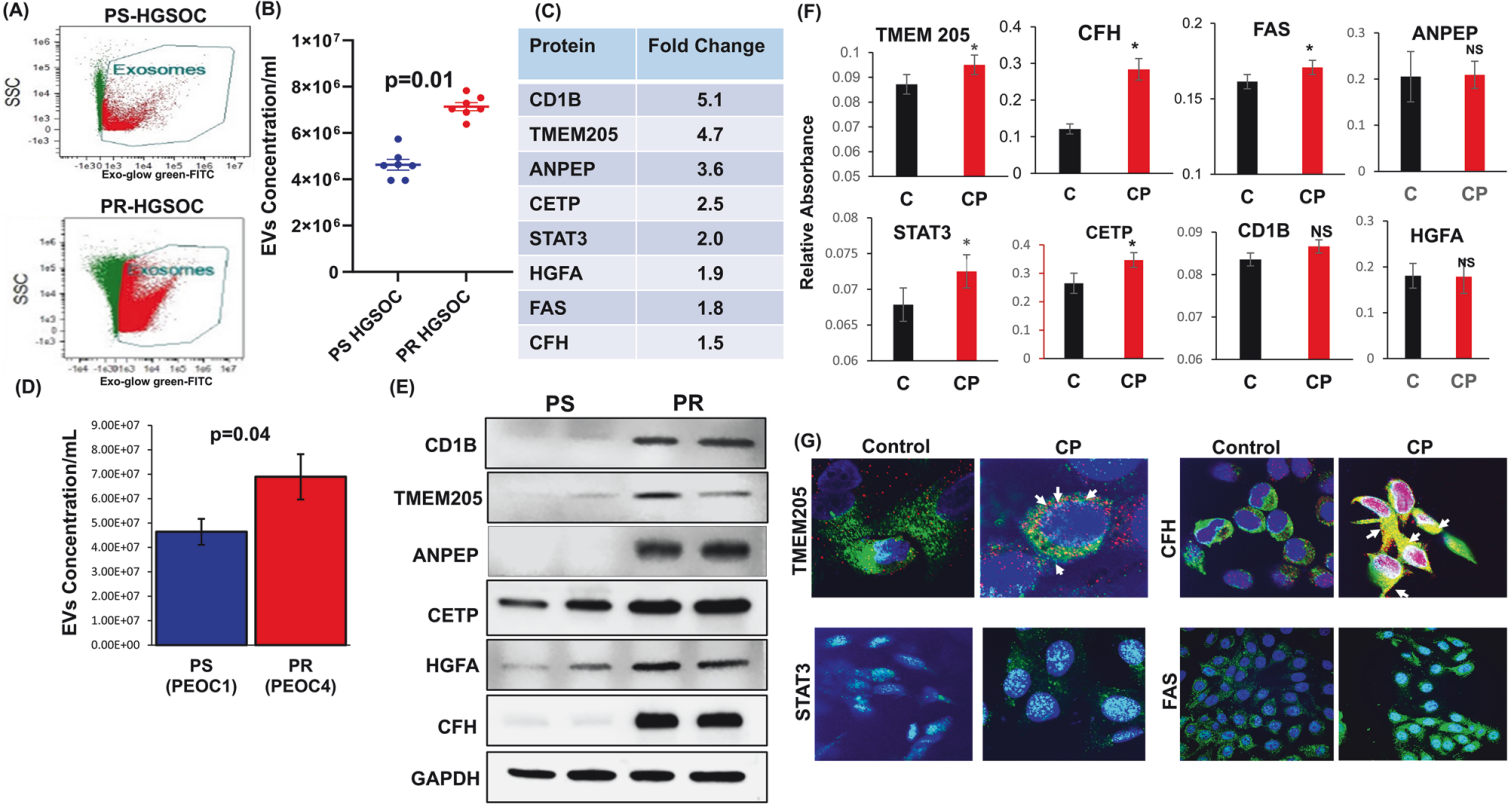
Increased EVs secretion with proteins in platinum-resistant HGSOC. **A** Image Stream Flow Cytometry (ISF) demonstrating the isolation of extracellular vesicles (EVs) using Exo-quick kit and SEC column in patient serum with platinum-sensitive (PS) and platinum-esistant (PR) high-grade serous ovarian carcinoma (HGSOC). **B** ISF-based quantification of EVs in both PS and PR HGSOC patient serum samples (*n* = 6, *p < 0.01). **C** Protein analysis of EVs from platinum-sensitive (PS) and platinum-resistant (PR) HGSOC patient serum samples using LC-MS/MS (*n* = 6). Candidate proteins were selected based on differential expression with statistical significance (p* < 0.05) in PR-HGSOC compared to PS patients. **D** EVs isolation by ultracentrifugation in PS and PR cells and ISF-based quantification of EVs in PS (PEOC1) and PR (PEOC4) patient derived cell lines (*n* = 5, *p* < 0.04). **E** Confirmation of selected candidate protein expression by western blot in two distinct patient-derived cell lines representing PS (PEOC1) and PR (PEOC4) phenotypes. **F** Carboplatin treatment enhances candidate protein expression on the membrane of platinum-resistant HGSOC cells in vitro. A significant increase in the expression of TMEM205, CFH, FAS, STAT3 and CETP in platinum-resistant cells (TR127) following a 24-hour carboplatin treatment, as determined by ELISA (*n* = 4, *p < 0.05). **G** Visualization of candidate protein localization in HGSOC platinum-resistant cells (TR127) treated with carboplatin for 24 h, using confocal microscopy.

EVs were successfully isolated from the serum of PS- and PR-HGSOC patients using the Exo-quick kit for the initial protein evaluation. For further EV isolation, ultracentrifugation and SEC column was utilized. EV isolation was confirmed by morphology using transmission electron microscopy (TEM) and by specific protein markers (CD9, CD63, CD81 and TSG101) (Supplementary Fig. 1A, B). The isolated extracellular vesicles (EVs) had an average size just above 100 nm, as quantified using imaging flow cytometry (ISF) (Supplementary Fig. 1C); a negative control illustrating size distribution is also shown (Supplementary Fig. 2A). The EV size distribution was gated based on the normalized frequency of fluorescent beads for imaging flow cytometry analysis, EV size was limited 30-120 nm (Supplementary Fig. 2B). Quantitative analysis of EV concentration, as determined by Image stream flowcytometry (ISF), revealed a statistically significant increase in EV concentration in PR-HGSOC patient serum samples when compared to PS counterparts (Fig. 1A, B, Supplementary Fig. 3A, B).

Isolated EVs from the serum of PS- and PR-HGSOC patients underwent a comprehensive proteomic analysis using Liquid Chromatography-Mass Spectrometry (LC-MS/MS). This analysis revealed elevated expression of several proteins in PR-HGSOC samples, relative to PS-HGSOC samples as measured by fold-change in expression with statistical significance (*p* < 0.05) (Fig. 1C). These “candidate” EV protein biomarkers for platinum resistance include transmembrane glycoprotein CD1B, transmembrane protein 205 (TMEM205), alanyl aminopeptidase (ANPEP), cholesteryl ester transfer protein (CETP), signal transducer and activator of transcription 3 (STAT3), hepatocyte growth factor activator (HGFA), fatty acid synthase (FAS), and complement factor-H (CFH).

### In-vitro and in-vivo evaluation of candidate EV protein expression after platinum therapy

Our in vitro model of HGSOC confirmed a significant increase of EV secretion in PR cells compared to PS (Fig. 1D, Supplementary Fig. 3C, D). We also confirmed the expression of candidate proteins CD1B, TMEM205, ANPEP, CETP, HGFA and CFH in PR-HGSOC cells and compared to PS cells with western blot (Fig. 1E). To assess how expression of the candidate EV proteins is affected by exposure to platinum therapy, we evaluated protein expression both in cell culture and in an orthotopic HGSOC mouse model. The in vitro model demonstrated increased expression of the candidate EV proteins in PR-HGSOC cells after treatment with carboplatin as measured by ELISA and visualized by immunofluorescence analysis (Fig. 1F, G, Supplementary Fig. 4). These results indicate an association between carboplatin exposure and the upregulation of the candidate proteins, underscoring their potential relevance in platinum resistance mechanisms.

**Fig. 2:**
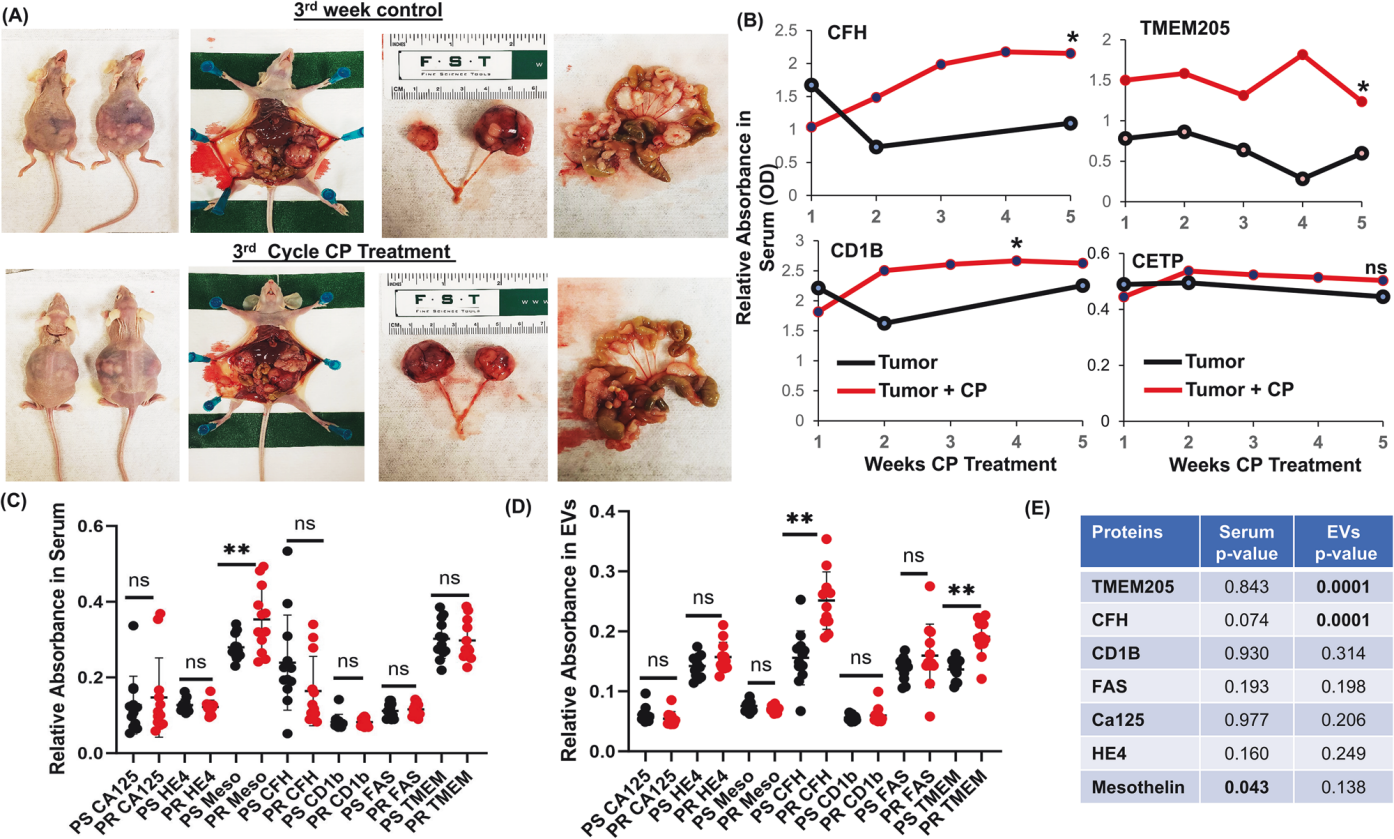
Identification of extracellular vesicle (EV) candidate expression during early carboplatin treatment in HGSOC mouse models. **A** BALB/c nude mice were intrabursal-injected with platinum-resistant HGSOC cells (TR127), recapitulating the clinical presentation of HGSOC with aggressive ovarian tumor growth and metastatic spread to the mesentery, diaphragm, and pelvic regions. After tumor growth confirmation at the 2nd week, intraperitoneal carboplatin (CP, 2 mg/kg) treatment was administered for 5 weeks. **B** Mouse serum samples were collected at the end of each treatment cycle before the subsequent CP cycle, and EV protein expression was assessed using ELISA (*n* = 4; **p ≤ 0.005; *p ≤ 0.01 compared to pre-CP treatment or the 1st cycle of CP treatment). **C**–**E** Evaluation of candidate proteins TMEM205, CFH, CD1B, and FAS with established biomarkers CA125, HE4 and Mesothelin in HGSOC patient serum and serum-derived extracellular vesicles (EVs) using ELISA. This analysis was conducted on samples from both PS and PR samples in a training cohort of *n* = 12 patients, ** revealing statistically significant differences (*p* < 0.001).

We then corroborated these findings using an in vivo orthotopic HGSOC mouse model. PR mice were developed. Platinum based intraperitoneal chemotherapy was administered to half of the mice on a weekly basis, the other mice were kept as controls. A total of five cycles were administered. At each cycle of chemotherapy, mice from both groups were sacrificed, serum collected and EVs isolated. Candidate EV protein analysis was performed by ELISA. This model revealed a statistically significant increase in the expression of a subgroup of candidate EV proteins (TMEM205, CFH and CD1B) (Fig. 2A, B, Supplementary Fig. 5). The remaining candidate EVs proteins (CETP, ANPEP, STAT3 and FAS) were not statistically significant in the CP treatment mice (Supplementary Fig. 6). This in vivo model gives further evidence that a subset of the identified candidate EV proteins hold promise as biomarkers for platinum resistance. These data also imply a potential upregulation of resistance mechanisms in the context of PR HGSOC.

### Evaluation of candidate protein expression in PS- and PR-HGSOC patient serum

Based on the in vitro and in vivo screening of predictive biomarkers, CFH, CD1B, and TMEM205 were selected as the most promising EV biomarkers for platinum resistance that warrant further investigation. In addition to these novel biomarkers, we also evaluated the established biomarkers for HGSOC, including CA125, HE4, and Mesothelin. We evaluated these biomarkers in an independent set of serum samples from patients with PS (*n* = 44) and PR-HGSOC (*n* = 44). Patient demographics and cancer stage, histology and treatment setting can be found in Supplementary Fig. 7. Protein analysis by ELISA in whole serum (*n* = 12) demonstrated no significant difference in candidate proteins between PS and PR samples, mesothelin was found to have a significant difference (Fig. 2C). In contrast, in EVs isolated from the same samples, expression of CFH and TMEM205 were significantly higher in PR patients (*n* = 12) (Fig. 2D) indicating the candidate proteins CFH and TMEM205 are increased significantly in EVs as opposed to whole serum. Among the established biomarkers, only mesothelin was found to be statistically different between PS and PR patients and this difference was only identified in whole serum but not in EVs (Fig. 2E). There was not a statistical difference in CA-125, HE4 or other candidate proteins such as CD1B and FAS, between PS and PR patients (either whole serum or EVs).

**Fig. 3:**
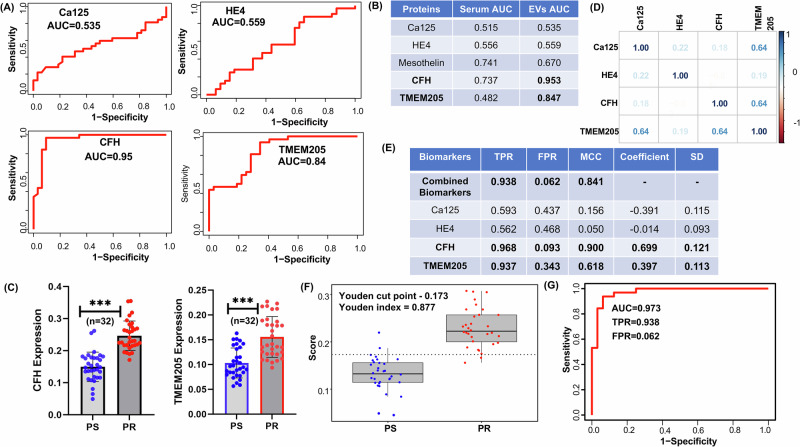
Identifying the sensitivity and specificity of EVs candidate proteins TMEM205 and CFH. **A**, **B** Receiver Operating Characteristic (ROC) curves depicting the diagnostic performance of CA125, HE4, CFH, and TMEM205, as determined by ELISA results from EVs isolated from serum samples of platinum-sensitive and platinum-resistant HGSOC patients (*n* = 32). Our candidate proteins, CFH and TMEM205, exhibited an impressive Area Under the Curve (AUC) exceeding 0.95 and 0.84, in contrast to CA125 and HE4 with an AUC of 0.53 and 0.55, respectively, in EV samples. Serum sample AUCs are also presented in (**B**). **C** Validation of candidate protein expression (CFH and TMEM205) in EVs derived from serum samples of platinum-sensitive and platinum-resistant HGSOC patients using ELISA (*p* < 0.0001). **D** Correlation plot of considered biomarkers (CFH, TMEM205, CA125 and HE4) across all patients. **E** Evaluation of out-of-sample prediction performance for PS and PR HGSOC patient samples. The optimal biomarker combination, determined by maximizing the smoothed empirical estimate of utility (SHUM), alongside the performance (true positive rate (TPR), false positive rate (FPR), and Mathew’s correlation coefficient (MCC)) of individual biomarkers through leave-one-out (LOO) analysis in the ELISA dataset, is presented. The coefficients of biomarkers within the optimal combination for enhanced diagnostic accuracy are detailed, with bootstrap standard errors (from 500 samples). **F** Boxplots of the optimal combination vector scores are shown across the outcome categories for all patients. Optimal cut point = 0.173 and Youden index is 0.877 (identified as PR if score is greater than cut-off). **G** Receiver operating characteristic (ROC) curve for diagnosing PR HGSOC via optimally combined biomarker (AUC = 0.973; TPR = 0.938; and FPR = 0.062).

These identified statistically significant candidate proteins CFH and TMEM205 were then validated in an additional independent set of 32 serum samples from patients with PS- and PR- HGSOC. Both EV proteins and proteins from whole serum were assessed using ELISA. Receiver Operating Characteristic (ROC) curve analysis was conducted to evaluate the diagnostic performance of the identified candidate protein biomarkers for platinum resistance in HGSOC. As seen in (Fig. 3A), both EV CFH and TMEM205 were found to have excellent diagnostic performance based on ROCs with area under the curve (AUC) values of 0.95 and 0.84, respectively. The diagnostic performance of CA-125 and HE4 was evaluated, which revealed poor diagnostic accuracy AUC values of 0.53 and 0.55 for platinum resistance in both EV and whole serum (Fig. 3A, B). In both cases, the AUC of the EV CFH and TMEM205 was significantly higher than that of whole serum (both with *p* < 0.0001) (Fig. 3B, C). Supplemental Figs. 8–10 demonstrate the differential expression of candidate proteins in PS- versus PR-HGSOC samples, both in EVs and whole serum. The high diagnostic performance of EV CFH and TMEM205 suggests their potential as non-invasive biomarkers for the detection of platinum resistance in HGSOC. The marked difference in the AUC values for these proteins between whole serum samples and EV-isolated samples underscores the importance of extracellular vesicles as a matrix for biomarker discovery. The modest performance of other candidate and established proteins in this analysis suggests that while they may contribute to a biomarker panel, they lack the robustness of CFH and TMEM205 as standalone indicators.

### Finding the optimal biomarker combination using the SHUM method

To determine the optimal combination of proteins for a potential biomarker panel, we evaluated the correlation between and within the novel candidate proteins and established protein biomarkers. The results indicate generally weak correlations between these proteins (Fig. 3D). We then integrated CFH, TMEM205 and CD1b with the established biomarkers CA-125 and HE4 for analysis. The optimal combination biomarker was derived by maximizing the corresponding smoothed approximation of the empirical hypervolume under manifolds (SHUM). To ensure the identifiability of the combination vector, we constrain the Euclidean norm of the biomarker coefficients to be 1. We employed the optimization algorithm proposed in Das et al. [30] to maximize the SHUM criterion under this constraint. We present the overall true positive rate (TPR), false positive rate (FPR), and Mathew’s correlation coefficient (MCC) for both the combined biomarker approach and individual biomarkers in Fig. 3E and Supplementary Fig. 11. Our results demonstrate that the diagnostic performance for platinum resistance in HGSOC of the combined biomarkers surpasses that of any individual biomarker. Although the diagnostic performance of CFH alone is promising, this is likely exaggerated by the limited sample size and would be less robust for further validation as compared to the combined biomarker. The calculated optimal cut-off point for PR, determined using Youden’s index, is 0.877 (Fig. 3F). As such, our proposed diagnostic rule for future use is as follows: if the combined biomarker value exceeds 0.877, the patient would be identified as PR; otherwise, the patient would be identified as negative for PR. The ROC curve for the combined biomarker shows excellent diagnostic capability with AUC = 0.973; TPR = 0.938; FPR = 0.062 (Fig. 3G).

## Discussion

Platinum resistance in the clinical course of ovarian cancer is a poor prognostic indicator [31]. This study presents a promising advancement in the pursuit of biomarkers for platinum resistance in HGSOC. We investigated the protein content of EVs derived from HGSOC cells using platinum-resistant cell lines, mouse models, and patient samples. Notably, we found that EVs from platinum-resistant ovarian cancer cells are enriched with a subset of proteins when compared to EVs from platinum-sensitive ovarian cancer cells. Out of larger subset of candidate biomarker proteins our studies identified, TMEM205 and CFH were found to have the most prominent and consistently increased expression in platinum-resistant ovarian cancer. Our findings underscore the diagnostic potential of proteins derived from EVs by demonstrating superior sensitivity and specificity as compared to analysis performed on whole serum samples and clinically utilized serum markers such as CA125 and HE4. The elevated expression of these EV proteins in PR-HGSOC, as demonstrated by our in vitro, in vivo, and serum analyses, aligns with the growing body of literature that recognizes the role of EVs in cancer progression and chemoresistance. In addition, our data supports that platinum-resistant patients have an increased abundance of EVs in their serum [20, 27, 32–34].

Previous studies have attempted to identify biomarkers of chemoresistance in ovarian cancer, such as Multi-drug resistance-associated proteins and p27, based on molecular mechanisms associated with platinum resistance or the elevation of membrane glycoproteins in patient serum samples [35, 36]. However, these studies are limited by the small number of ovarian cancer patient samples and were not limited to high-grade serous histology. These biomarkers lacked any further validation. In the current study, we were able to demonstrate a marked superiority in the diagnostic accuracy of our biomarker proteins, CFH and TMEM205 within EVs, when compared to whole serum. This is a novel discovery that emphasizes the potential of EVs as a novel matrix for biomarker discovery in HGSOC. This is particularly relevant given the limitations of CA125 and HE4 to predict response to platinum therapy, which our study further demonstrates by poor sensitivity and specificity for platinum resistance. Further, our study provides evidence that the top candidate EV proteins we evaluated, TMEM205 and CFH, are significantly elevated early in the treatment regimen in PR-HGSOC mouse models. This suggests that the expression of these proteins could serve as an early indicator of platinum resistance in HGSOC. These findings are consistent with previous reports identifying membrane proteins as promising early prognostic and predictive biomarkers [19, 37–39].

Recent publications have demonstrated increased expression of TMEM205, and proteins associated with the EV secretion pathway such as Rab7 and Rab27a in platinum-resistant HGSOC cells [26, 27, 40, 41]. These findings indicate that the upregulation of these proteins may contribute to the molecular mechanisms that lead to chemoresistance and recurrence. TMEM205, a transmembrane glycoprotein, is particularly noteworthy due to its diverse functions and interactions with various proteins which have implications for different cancer types, including ovarian cancer. We have previously shown TMEM205 as a therapeutic target, making these EV proteins notable as both prognostic and predictive biomarkers [26]. Our studies build on the growing knowledge of the intricate molecular mechanisms underpinning platinum resistance in HGSOC.

The high diagnostic performance of CFH and TMEM205 in EVs in the current study suggests their utility as part of a non-invasive biomarker panel for platinum resistance in HGSOC. Although further studies will evaluate the individual performance of each biomarker alone, we feel the combined panel has the most promise for validation. This could potentially guide clinicians in tailoring treatment plans more effectively, and potentially avoid futile and toxic therapy. This has implications for clinical practice, as it not only allows for the earlier identification of patients for prognostic purposes, but also enables a more patient-specific chemotherapy approach (i.e. precision medicine). Such approaches could theoretically include adding an additional non-platinum agent, switching to a non-platinum regimen, or including a novel agent to improve platinum sensitivity in the context of a clinical trial. The unique EV protein profiles associated with platinum resistance may serve as potential therapeutic targets, such as targeting TMEM205 with DAP compounds [26]. Regardless of intervention, detecting platinum resistance as early as possible is invaluable as it informs important treatment decisions, could guide patient participation in clinical trials, and minimizes unnecessary toxicity.

### Limitation of this study

While our study provides promising insights, there are several limitations to the current data. The use of cell lines and mouse models may not fully replicate human disease dynamics or fully illustrate tumor heterogeneity, and the retrospective analysis of patient serum samples may introduce biases. A risk of EV degradation with storage exists, although all samples analyzed were collected and stored from a similar time period. Our data are further limited by the relatively small number of patient samples collected from two different geographic area, limiting generalizability to different racial or ethnic groups. Our future research is focused on prospective evaluations of patient serum samples before and during chemotherapy. This will provide a more comprehensive understanding of the biomarkers’ dynamics and clinical relevance and potential impact on oncologic outcomes. Additionally, exploring the therapeutic potential of EV secretion inhibitors and inhibition of TMEM205 in reversing platinum resistance offers a promising avenue for future investigations.

In conclusion, our study highlights the potential of EV proteins, particularly CFH and TMEM205, as novel biomarkers for predicting platinum resistance in HGSOC. This discovery opens new pathways for prediction of clinical behavior and therapeutic strategies, providing opportunities to better understand and target mechanisms of chemoresistance for patients with HGSOC.

## Supplementary information

Supplementary Figure 1 to Figure 11

## Data Availability

The datasets utilized and/or analyzed during this study are available from the corresponding author upon reasonable request.
