# Supplementary material for: Circulating extracellular vesicles protein expression for early prediction of platinum-resistance in high-grade serous ovarian cancer
Source: Oncogene. 2025 Apr 10;44(17):1197–203. doi: 10.1038/s41388-025-03382-4 (PMC12015105; doi:10.1038/s41388-025-03382-4)
Supplement: Supplementary file 1 — Supplementary Figure 1 to Figure 11 [file 41388_2025_3382_MOESM1_ESM.pdf]

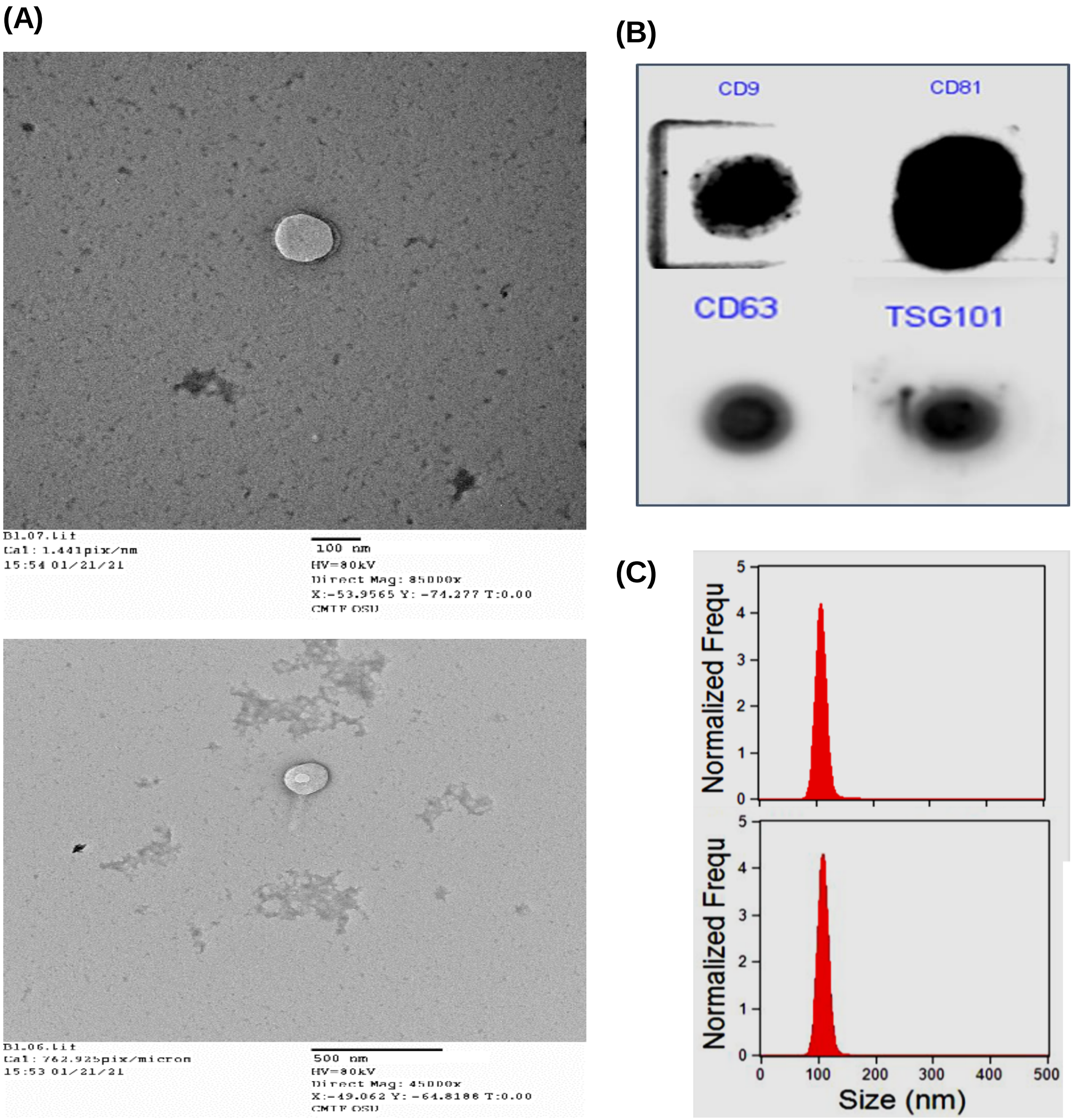

Sup Figure 1. A) Morphological characterization and size measurement of extracellular vesicles (EVs) (indicated by red arrows) using classical transmission electron microscopy (TEM). **B)** Validation of EV-specific markers CD9, CD63, CD81, and TSG101 through dot blot assay. **C)** Confirmation of EV size through flow cytometry in serum samples from ovarian cancer (OC) patients.

(A)

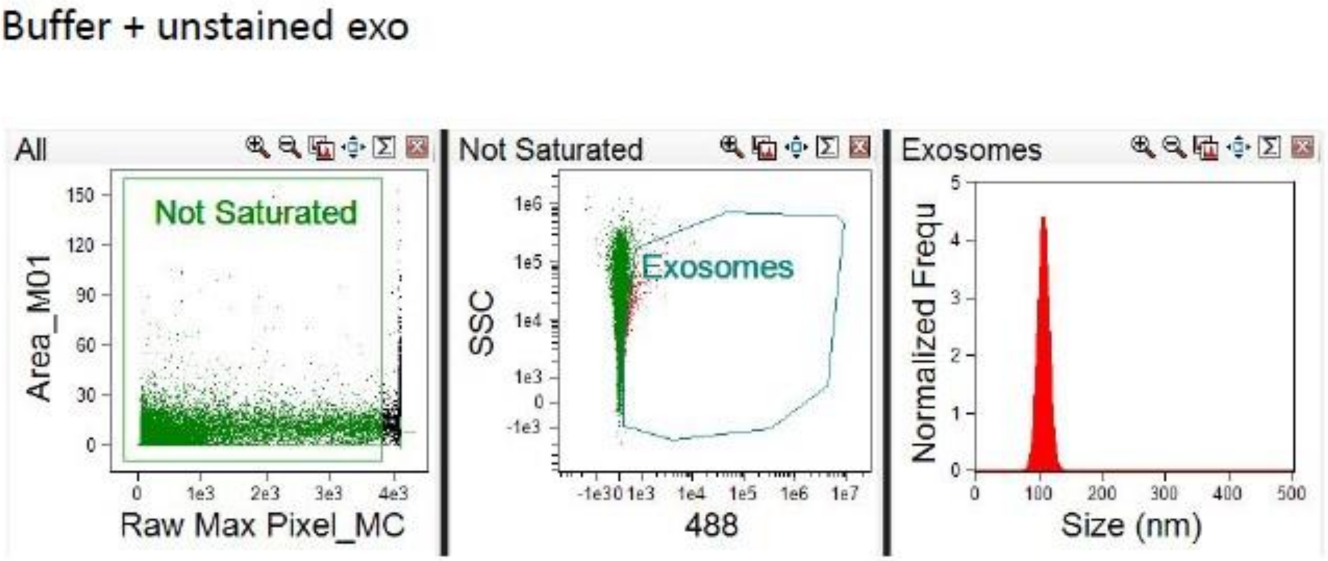

(B)

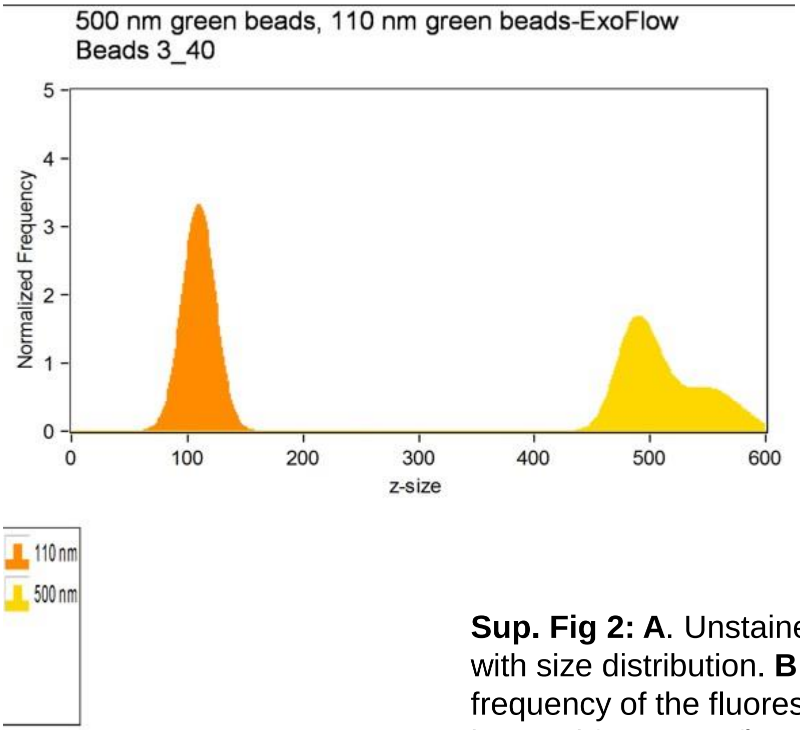

z-size

Population	Median
500 nm green beads	500
110 nm green beads	109.99

Sup. Fig 2: A. Unstained EVs data for negative control with size distribution. **B**. Based on the Normalized frequency of the fluorescent beads the EV size distribution is gated for Image flowcytometry.

(A)

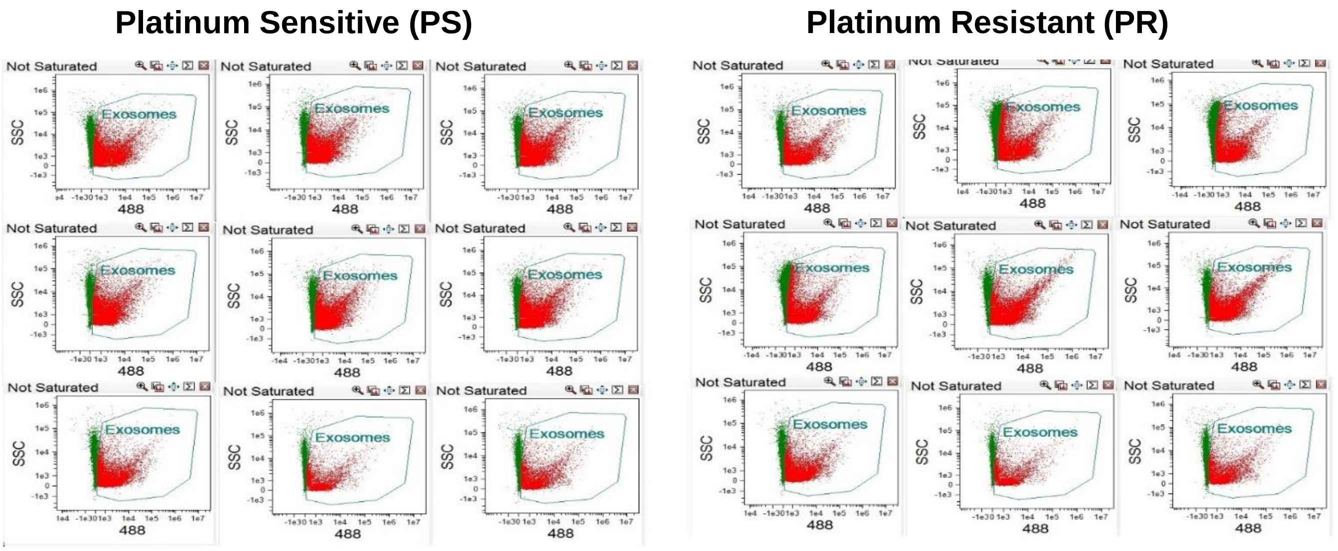

(B)

PS	PR
4636692	6891783
4584312	7519641
4938525	7284356
4593770	6375610
3955387	7037521
5739775	7831073

(D)

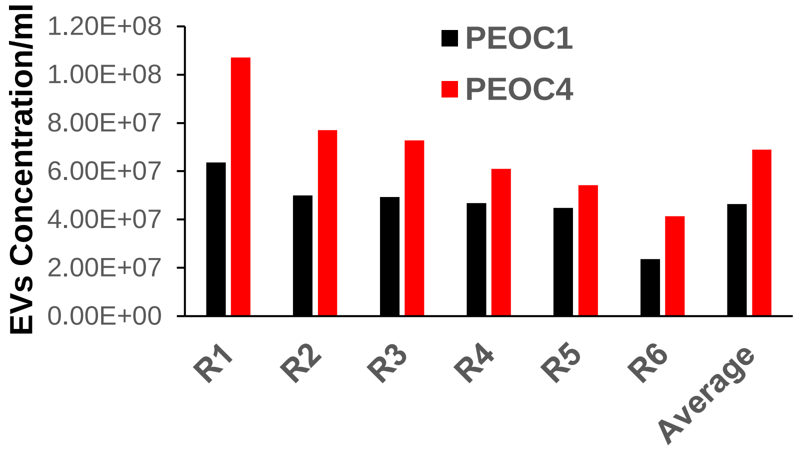

(C)

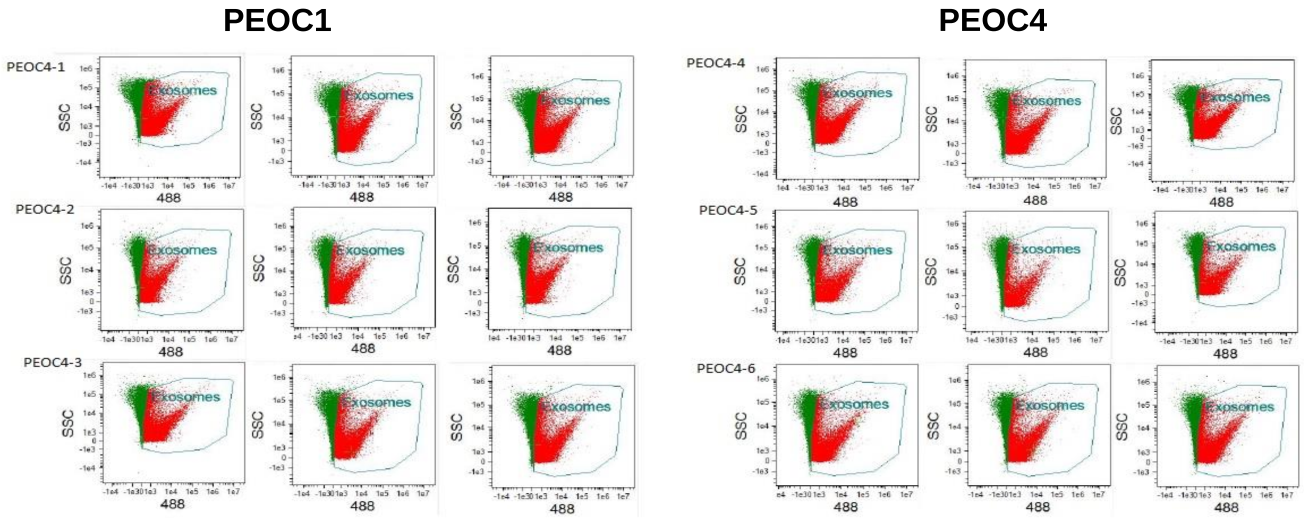

Sup Figure3. A & B). EVs isolated by microfluidic chip or column in HGSOC platinum sensitive (PS) platinum resistant (PR) samples. **C & D**) EV isolaton form platinum sensitive (PEOC1) and resistant (PEOC4) cell lines analysis using ultracentrifuge and EV quantification by image stream flow cytometry (ISF)**.**

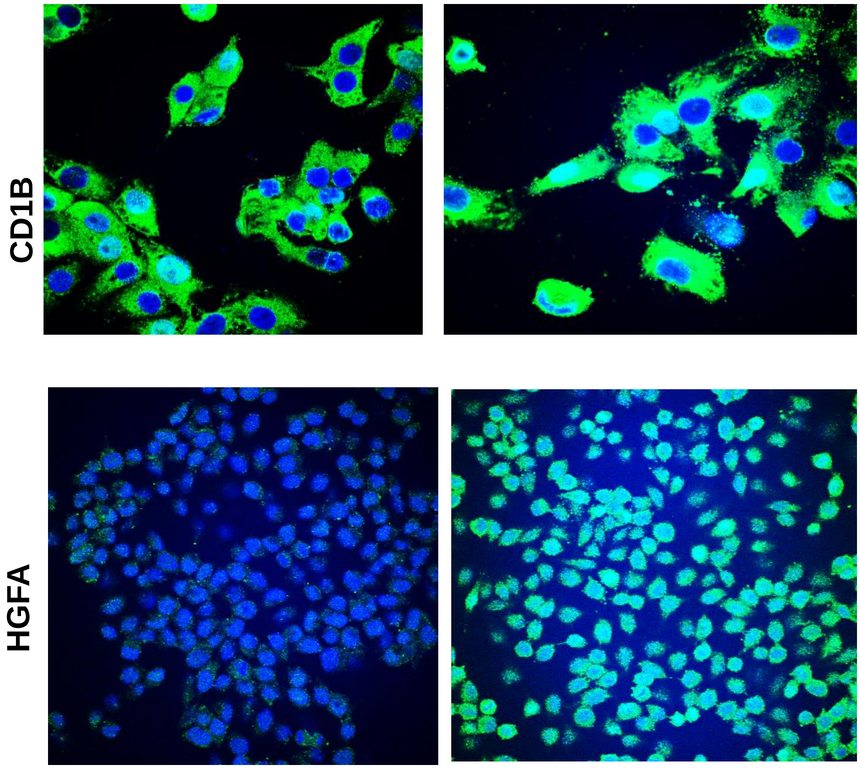

Sup Figure 4. Visualization of candidate protein localization in HGSOC platinum-resistant (TR127) cells treated with carboplatin for 24 hours, using confocal microscopy

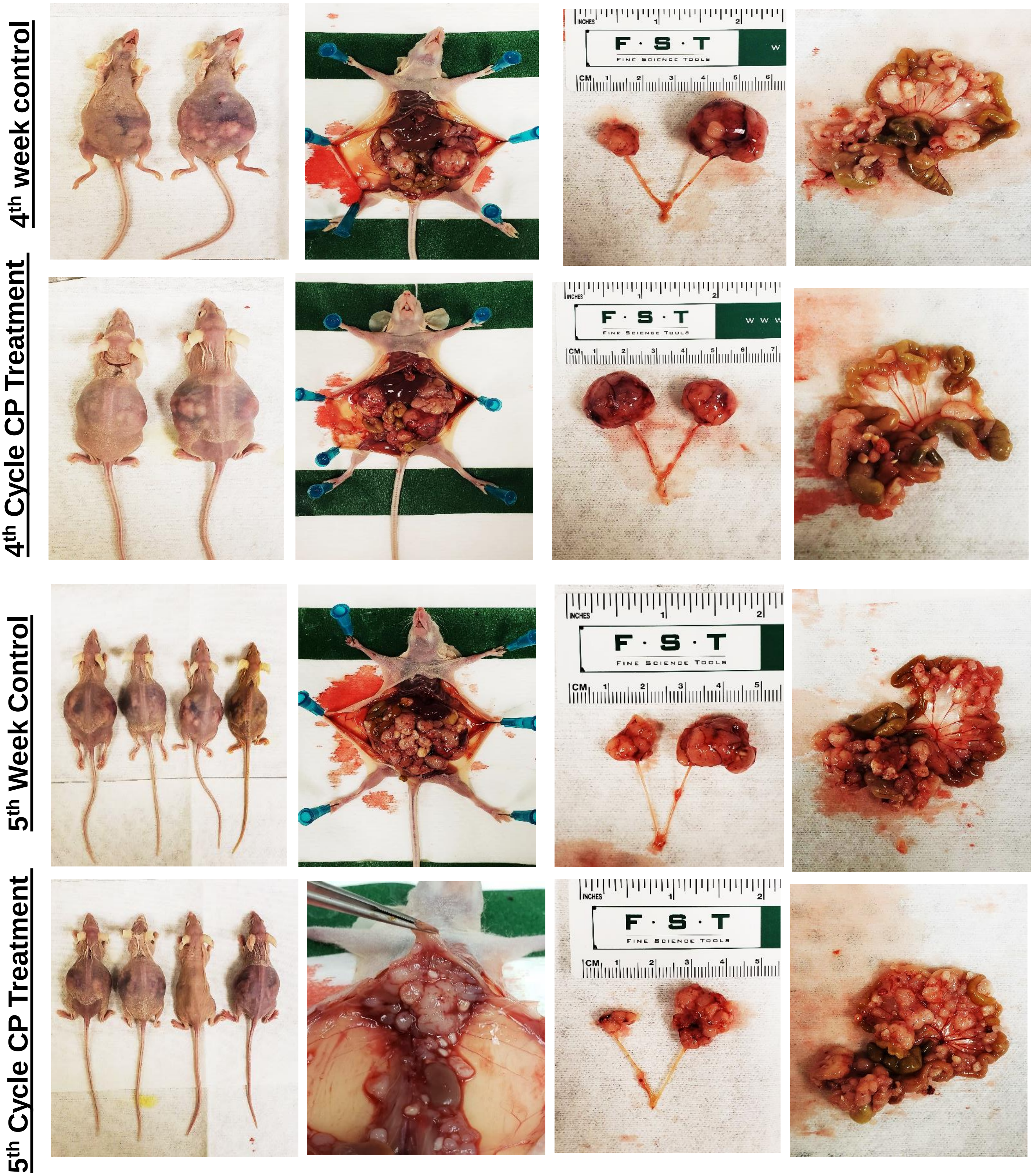

Sup Figure 5. Established orthotopic mouse models that accurately replicate the clinical distribution of disease in humans, with tumors originating from the Mullerian structures by injecting tumor cells into the ovarian bursa. Identified tumors metastasizing throughout the peritoneal cavity, including the bowel, mesentery, and diaphragm, in both untreated mice and those treated with carboplatin (2 mg/kg/week) for five cycles.

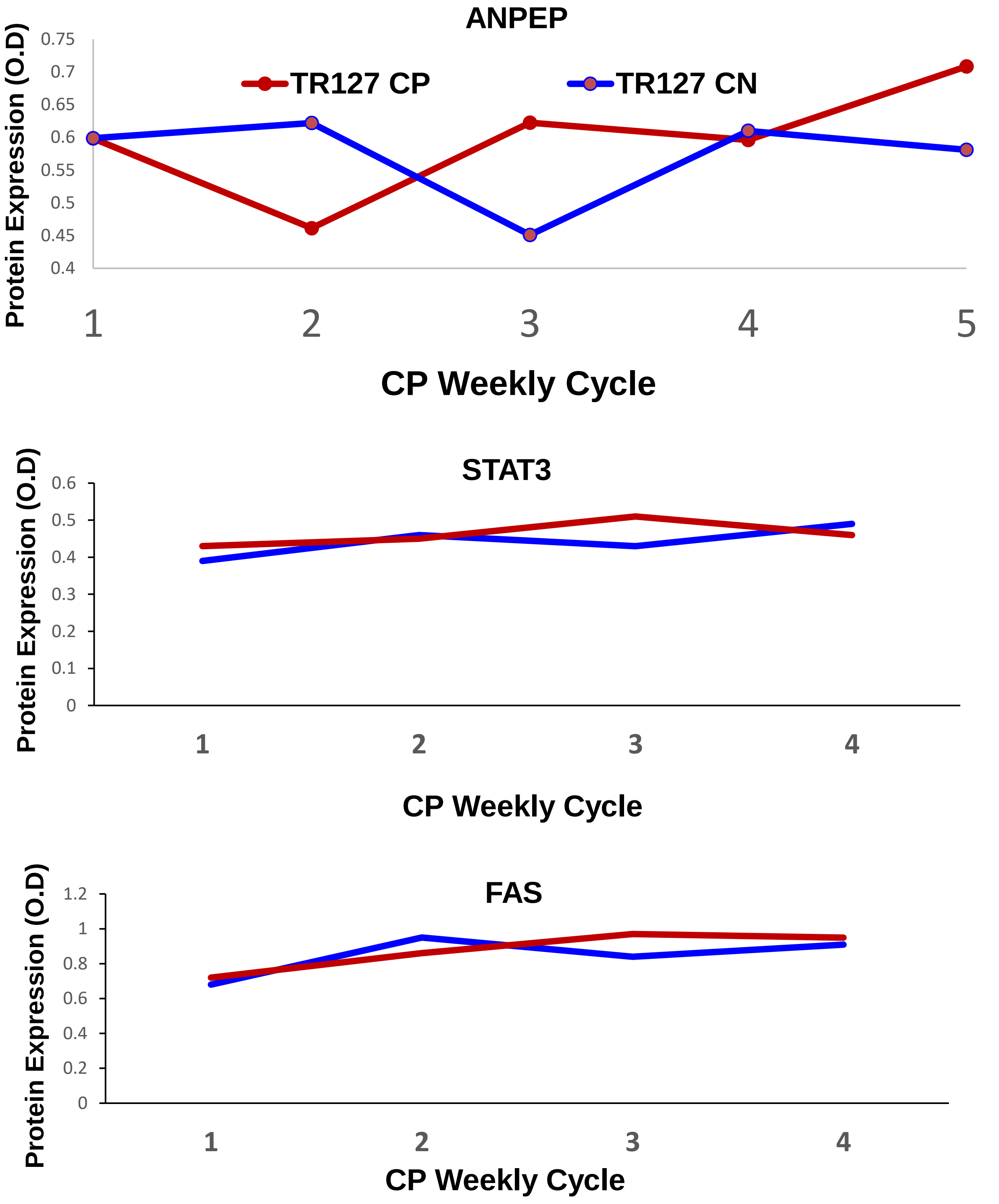

Sup. Figure 6. Serum samples were collected at the end of each treatment cycle before the subsequent CP cycle, and EV protein expression was assessed using ELISA (n=6).

Sup. Figure 7. Demographics of our patient groups (median age of groups, race, stages, histology and treatment setting.

	Platinum Sensitive N=42	Platinum Resistant N=42
**Demographics**		
**Age at Collection (years)**	63.4 ±9.7	60.6 ±10.5
**Race**		
**White**	38	40
**Black**	2	2
**Asian**	2	0
**Original Stage**		
**IIB**	2	1
**IIIA**	1	1
**IIIB**	0	0
**IIIC**	36	35
**IVA**	1	3
**IVB**	2	2
**Histology**		
**High Grade Serous**	42	42
**Phase of Treatment**		
**Primary**	25	3
**End of Treatment**	3	0
**Recurrent**	14	39

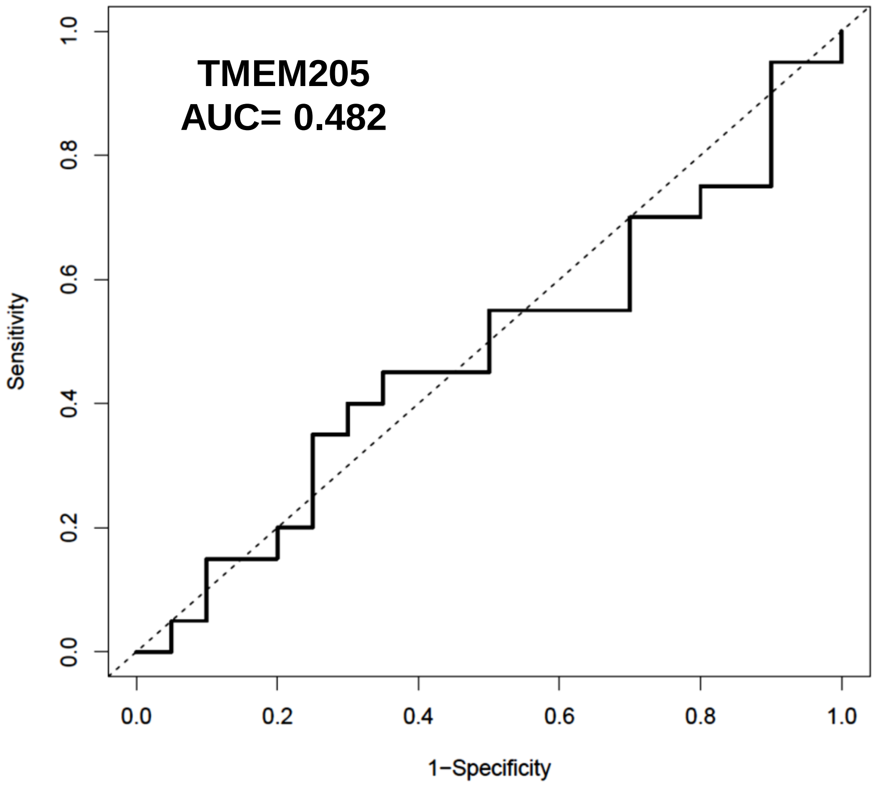

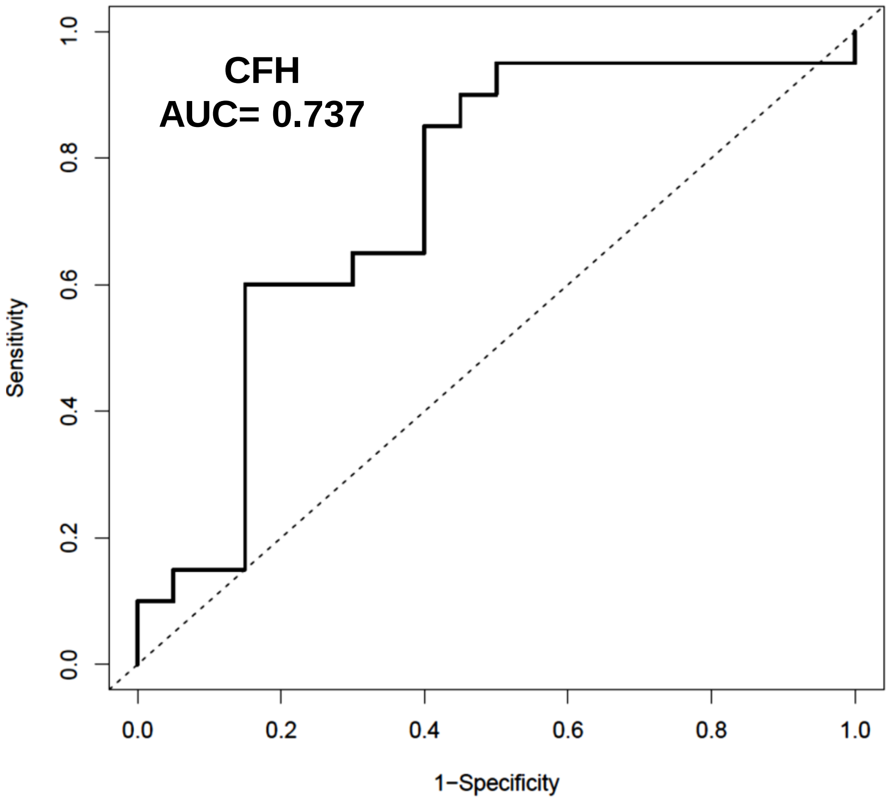

Sup Figure 8. Are under curve (AUC) illustrating the differential expression of candidate proteins TMEM205 and CFH, measured by ELISA, in a new set of serum samples from patients with high-grade serous ovarian cancer (HGSOC), categorized as platinum-sensitive (PS) and platinum-resistant (PR) (n=21).

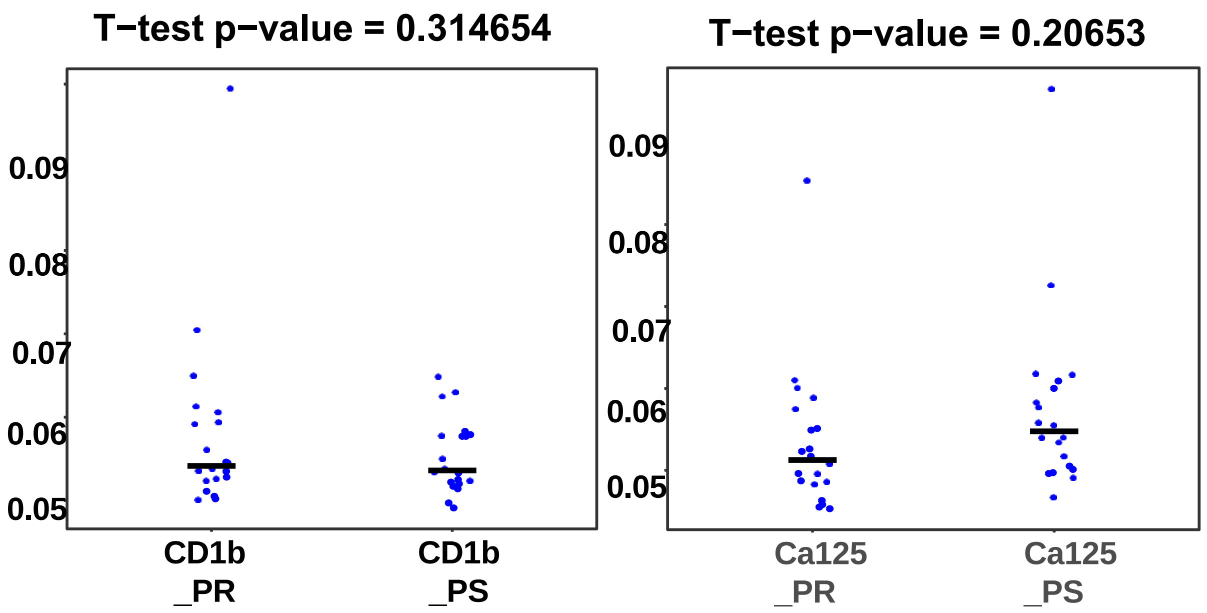

Sup Figure 9. Dot plot of differential expression of EVs proteins (CD1B and Ca125) as measured by ELISA in new set of HGSOC platinum sensitive (PS) and resistant (PR) patient samples (n=21).

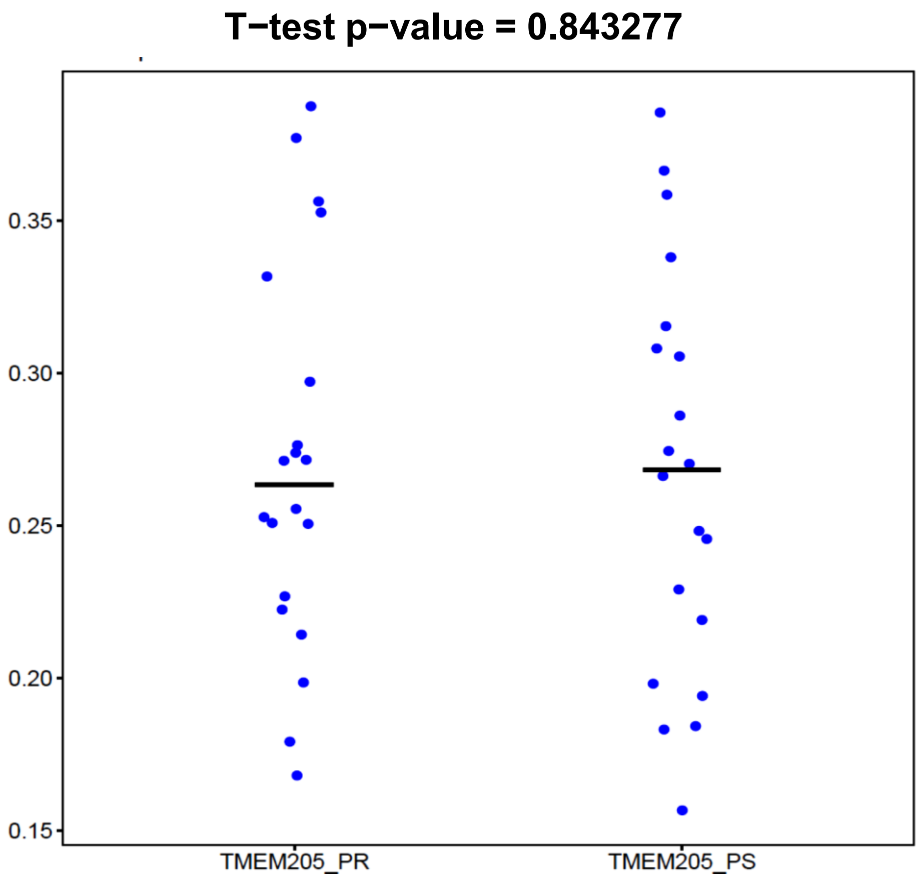

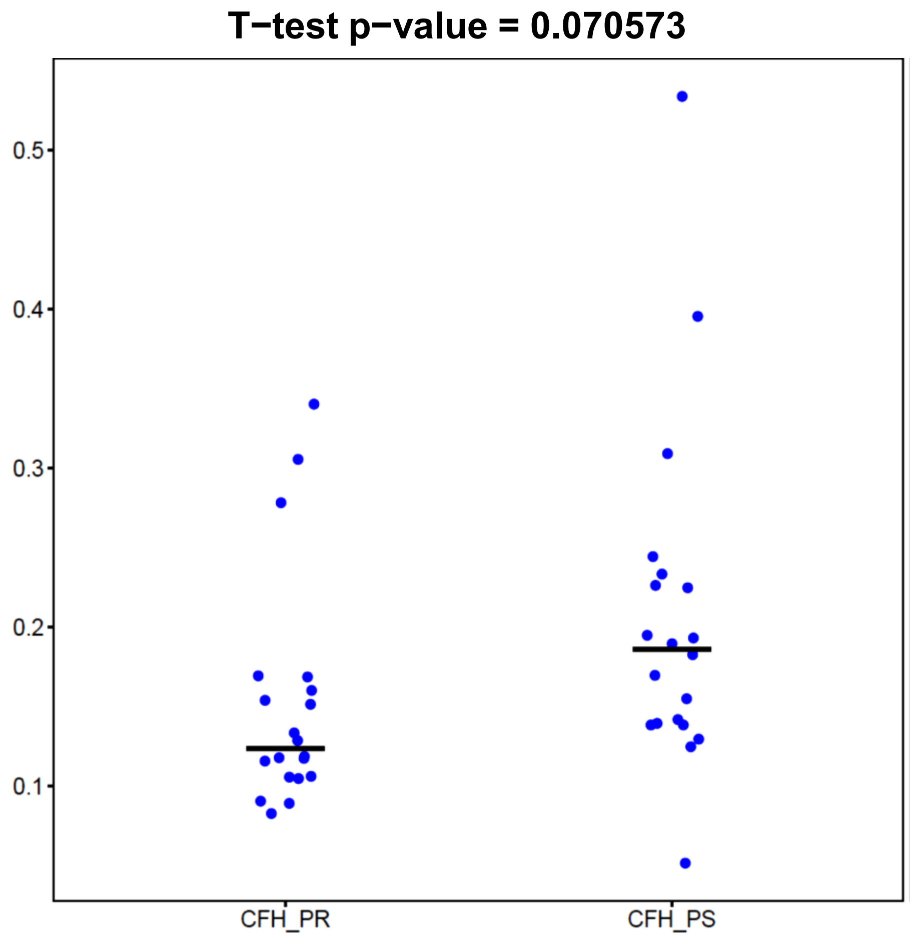

Sup Figure 10. Dot plot illustrating the differential expression of candidate proteins TMEM205 and CFH, measured by ELISA, in a new set of serum samples from patients with high-grade serous ovarian cancer (HGSOC), categorized as platinum-sensitive (PS) and platinum-resistant (PR) (n=21).

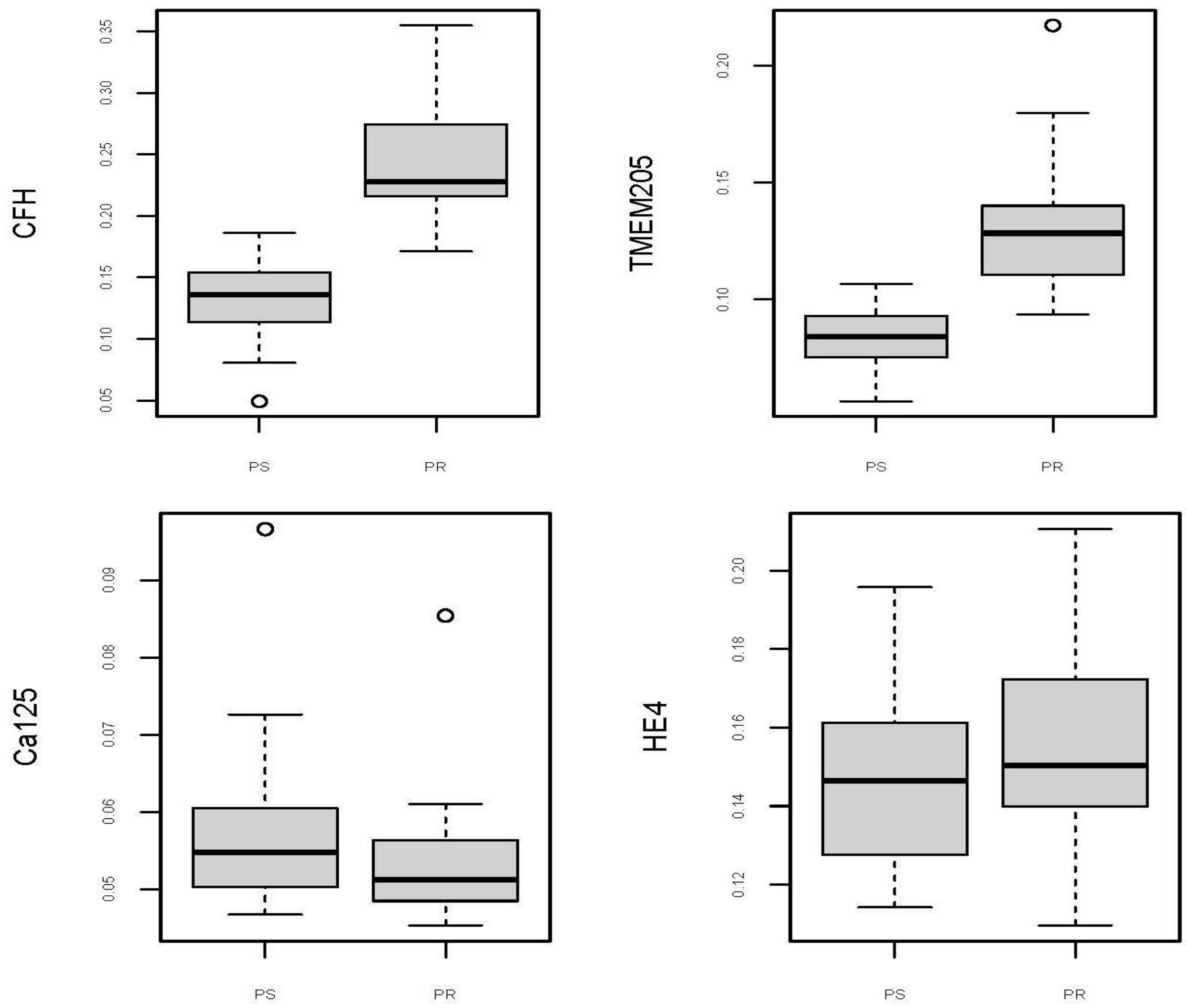

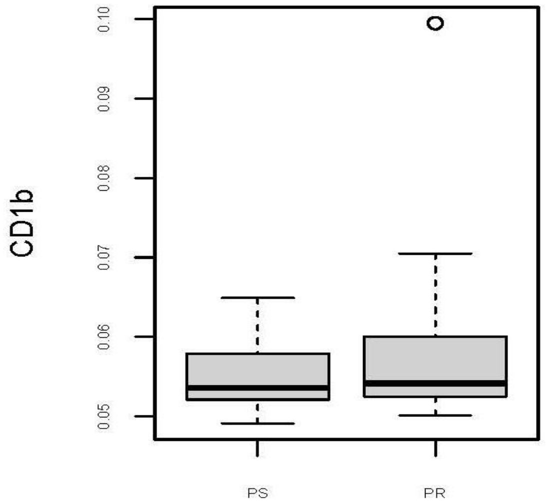

Sup Figure 11. Data from 5 protein biomarkers (Ca125, HE4, CFH, TMEM205, CD1b) were used to classify patient subgroups
(PS and PR). The optimization algorithm proposed by Das et al. (2022) was used to maximize an smoothed estimate of hyper-volume under manifolds (SHUM). We read in raw biomarker data from all patient groups, and subset it to two patient subgroups: PS and PR (n=21).
